# Severe multiple plant food allergy with LTP identification as melon allergen

**DOI:** 10.1186/2045-7022-1-S1-P1

**Published:** 2011-08-12

**Authors:** Anna Sokolova, Borja Bartolomé, Graca Sampaio, Maria Ines Mascarenhas, Helena Carreiro

**Affiliations:** 1 Hospital Fernando Fonseca, Amadora, Portugal; 2 Bial-Aristegui R&D Department, Bilbao, Spain

## Introduction

Allergy to plant-derived foods is variable in clinical presentation and in dangerousness due to properties of allergens involved. Clinical data permits to identify some markers of allergens involved and laboratory investigation completes this data.

## Clinical case

Caucasian 16-year-old boy was refered to Immunoalergology Unit for food allergy investigation. Since early childhood the patient avoids ingestion of peach although without any symptoms to mention. At the age of 7 he experienced labial and palpebral angioedema, oropharyngeal pruritis and dyspnoea immediately after ingestion of a non-peeled fresh apple and later with a cooked apple. At 8 years of age he experienced a similar reaction after ingestion of a melon. In the exposition to peanut powder he experiences nasal and oropharyngeal pruritis. The skin prick tests performed with extracts of common aeroallergens were negative. The skin prick tests were positive with extracts of peach, apple, melon, peanut, walnut, hazelnut and almond. Specific IgE was elevated for peach peel and pulp, apple and melon peel, walnut, raw and roasted peanut, hazelnut and for purified peach LTP. The molecular mass of the IgE binding bands was calculated by SDS PAGE immunoblotting with identification of 15-17 kDa bands in all the extracts assayed (apple, melon and peach peel and walnut). Immunoblotting inhibition method was performed with apple peel extract in solid phase and purified LTP (Pru p 3) in inhibition phase with a total IgE binding inhibition. The proteomic study confirmed LTP nature of melon protein involved in allergic reaction. The patient had an indication of a strict avoidance diet (rosaceae fruits, melon, peanut, tree nuts) and use of IM epinephrine kit.

## Discussion

We present a clinical case of severe allergy to botanically unrelated, plant-derived foods. LTP involvement was suggested by a clinical data and confirmed by EAST (elevated specific IgE to purified peach LTP), SDS Page immunoblotting inhibition and proteomic study. To our knowledge there are no previous descriptions of LTP proteins involved in allergy to melon.

